# Acute Kidney Injury in Pregnancies Complicated With Preeclampsia or HELLP Syndrome

**DOI:** 10.3389/fmed.2020.00022

**Published:** 2020-02-07

**Authors:** Jamie Szczepanski, Ashley Griffin, Sarah Novotny, Kedra Wallace

**Affiliations:** ^1^Department of Obstetrics and Gynecology, University of Mississippi Medical Center, Jackson, MS, United States; ^2^Program in Neuroscience, University of Mississippi Medical Center, Jackson, MS, United States; ^3^Department of Neurobiology and Anatomical Sciences, University of Mississippi Medical Center, Jackson, MS, United States

**Keywords:** renal injury, hemolysis elevated liver enzymes low platelet count (HELLP), preeclampsia, chronic kidney disease (CKD), acute kidney injury (AKI)

## Abstract

Acute kidney injury that occurs during pregnancy or in the post-partum period (PR-AKI) is a serious obstetric complication with risk of significant associated maternal and fetal morbidity and mortality. Recent data indicates that the incidence of PR-AKI is increasing, although accurate calculation is limited by the lack of a uniform diagnostic criteria that is validated in pregnancy. Hypertensive and thrombotic microangiopathic disorders of pregnancy have been identified as major contributors to the burden of PR-AKI. As is now accepted regarding preeclampsia, HELLP syndrome and atypical hemolytic uremic syndrome, it is believed that PR-AKI may have long-term renal, cardiovascular and neurocognitive consequences that persist beyond the post-partum period. Further research regarding PR-AKI could be advanced by the development of a pregnancy-specific validated definition and classification system; and the establishment of refined animal models that would allow researchers to further elucidate the mechanisms and sequelae of the disorder.

## Introduction

Acute kidney injury during pregnancy (PR-AKI) is associated with rates of maternal mortality and fetal loss that range from 30 to 60%, making it a life-threatening event (1). PR-AKI was until recently believed to be a relatively rare and declining complication of pregnancy that was primarily associated with sepsis, complicated pregnancy terminations and residence in low-income countries (2, 3). The lack of uniform diagnostic criteria limits the ability to accurately determine the incidence of PR-AKI and to quantify its influence on morbidity and mortality. However, recent data suggests that the incidence of AKI is increasing. PR-AKI is associated with an increased risk of chronic kidney disease (CKD), hypertension and cardiovascular disease (4, 5). PR-AKI is commonly associated with hypertensive conditions of pregnancy which themselves are associated with increased risks of cardiovascular disease later in life (6). The scope of the current review will be to highlight the relationship between preeclampsia, HELLP syndrome, thrombotic microangiopathies affecting pregnancy, acute fatty liver of pregnancy and AKI and to review the data regarding progression of PR-AKI to CKD.

## Incidence of PR-AKI

Incidence of PR-AKI in the United States has been estimated to have increased from 2.3 to 4.5 per 10,000 deliveries between 1998 and 2008 (7). It has been estimated that the incidence of AKI attributed to obstetric causes is <1 in 20,000 pregnancies (8). Retrospective studies comparing the incidence of PR-AKI from 1998–1999 to 2008–2009 have shown that in the United States the incidence of PR-AKI has increased from 2.29/10,000 deliveries to 4.52/10,000 deliveries in this 10 year time period and from 0.48/10,000 deliveries to 2.17/10,000 deliveries in the same 10 year time period in postpartum women (7, 9). These results were similar to studies performed in Canada, which also provided evidence that the incidence of PR-AKI is increasing (10, 11). Other studies cite incidences between 50 and 61% of PR-AKI among critically ill obstetric patients with a direct correlation between disease severity and mortality (12, 13). The burden of PR-AKI remains greater in developing countries with the World Health Organization stating that the Maternal Mortality Ratio is 239/100,000 women in developing countries compared to 12/100,000 in developed countries (14).

A study looking at severe complications in pregnancy noted a 97.26% increase in acute renal failure cases occurring from 1998–1999 to 2008–2009, and a 351% increase in postpartum acute renal failure (ARF) cases between these time periods, highlighting the fact that PR-AKI can occur in the postpartum as well as antepartum periods (9). A 2016 retrospective cohort study sought to investigate the rise in obstetric ARF in the United States and noted a 10% yearly increase between the periods 1999–2001 and 2010–2011 (95% confidence interval [CI] 8–11%) (7). In addition to an increase in ARF, the authors also reported an increase in maternal mortality and in dialysis treatment, but there was a decrease in the overall severity of ARF (7). It has been estimated that the incidence of AKI attributed to obstetric causes is <1 in 20,000 pregnancies (8). Factors that have been hypothesized to contribute to the rise in PR-AKI include: increasing pregnancies among women of advanced maternal age (35 years or older), obesity, diabetes, chronic hypertension, multifetal gestation, cesarean delivery, previous cesarean deliveries, induction of labor, polyhydramnios, antepartum hemorrhage, placental abruption, or placenta previa, cardiac failure, lupus erythematosus, and CKD (7, 9). However, changes in the management of obstetric conditions or ascertainment of AKI could also be implicated.

## Diagnosing AKI in Pregnancy

In order to accurately determine the incidence of PR-AKI, uniform diagnostic criteria must be defined. Several classification systems have been developed to streamline research and clinical practice with respect to AKI in non-pregnant individuals. In 2004, the Acute Dialysis Quality Initiative (ADQI) group published the RIFLE criteria, in an attempt to create a uniform definition of AKI and to aid in the assessment of the spectrum of severity of AKI (Table 1) (15). In 2007, the Acute Kidney Injury Network (AKIN) published additional criteria to improve the sensitivity and specificity of AKI diagnosis as outlined in RIFLE (Table 1) (16, 17). In 2012 the Kidney Disease Improving Global Outcomes (KDIGO) released their clinical practice guidelines for AKI, which built off of the RIFLE criteria and the AKIN criteria. KDIGO defines AKI as any of the following:

Increase in serum creatinine by 0.3 mg/dL or more within 48 h
Increase in serum creatinine to 1.5 times baseline or more within the last 7 days
Urine output <0.5 mL/kg/h for 6 h.

**Table 1:** RIFLE (Risk, Injury, Failure, Loss of kidney function and End-stage kidney disease) classification and the AKIN Modification of the RIFLE classification.

**RIFLE CLASSIFICATION**	
**Class**	**Clinical picture**
Risk	1.5-fold 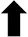 serum creatinine OR 25% 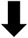 GFR OR UO <0.5 ml/kg/h for 6 h
Injury	2-fold 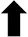 serum creatinine OR 50% 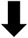 GFR OR UO <0.5 mL/kg/h for 12 h
Failure	3-fold 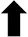 serum creatinine OR 75% 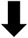 GFR OR UO <0.3 mL/kg/h for 24 h OR no urine output for 12 h
Loss of kidney function	Complete loss of kidney function (>4 weeks)
End-stage kidney disease	Complete loss of kidney function (>3 months)
**AKIN MODIFICATION**	
Absolute 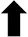 serum creatinine 0.3 mg/dL or more (>26.4 μmol/L)	
OR	
1.5-fold 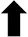 baseline serum creatinine	
OR	
Oliguria <0.5 mL/kg/h for >6 h; reduction in urine output	

The KDIGO has also recommended a staging system for the severity of the AKI (18). While these guidelines have assisted in the staging and classification of AKI, none of these criteria are validated for use in pregnancy.

There are several factors that make the diagnosis of AKI in pregnancy more challenging than in the non-pregnant state and assessment of renal function parameters used in non-pregnant individuals cannot always provide an accurate measurement in pregnancy. Due to the physiological changes and increase in glomerular filtration rate (GFR) a reduction of serum creatinine during pregnancy occurs, making the early and accurate diagnosis of AKI more difficult. This physiologic decrease in serum creatinine may mask early or mild changes in renal function. Comparison to baseline values is often not possible, as renal function parameters are often not obtained in pregnancy until injury is clinically suspected. Additionally, pregnant women may have a 30–40% reduction in GFR without significant increases in serum creatinine (19).

The American College of Obstetricians and Gynecologists (ACOG) defines renal insufficiency in the setting of hypertensive disorders of pregnancy as a serum creatinine level >1.1 mg/dL or a doubling of the serum creatinine concentration in the absence of renal disease (20). However, as of the time of this review there is no consensus on the diagnostic criteria that should be utilized for PR-AKI. In addition to impacting patient care, the lack of strict diagnostic criteria likely contribute to the variation in reported incidence of PR-AKI.

Outside of the clinical diagnostic criteria for AKI that relies heavily on serum creatinine levels, there has been an increased awareness for the need to identify AKI biomarkers. Among some of the circulating and urinary biomarkers that have shown some promise for clinical utility are neutrophil gelatinase-associated lipocalin (NGAL), liver-type fatty acid-binding protein (L-FABP), kidney injury molecule-1 (KIM-1), and cystatin C (21, 22). While several of these biomarkers have been reported to have great clinical utility, especially when used in conjunction with serum creatinine levels, a single biomarker for clinical diagnosis of AKI has yet to be identified. For biomarker utility during pregnancy, there's even more of a challenge as the biomarkers have to found to not be dependent upon weight, muscle mass or other pregnancy-related changes. For a full review of biomarkers in AKI, see Beker et al. (22). Several studies to date are investigating the clinical utility of using several of these proposed AKI biomarkers to gauge renal function and injury during pregnancy, especially those pregnancies complicated by hypertensive disorders, or to even predict high risk pregnancies (23–25).

## AKI During Pregnancy

**Figure 1:**
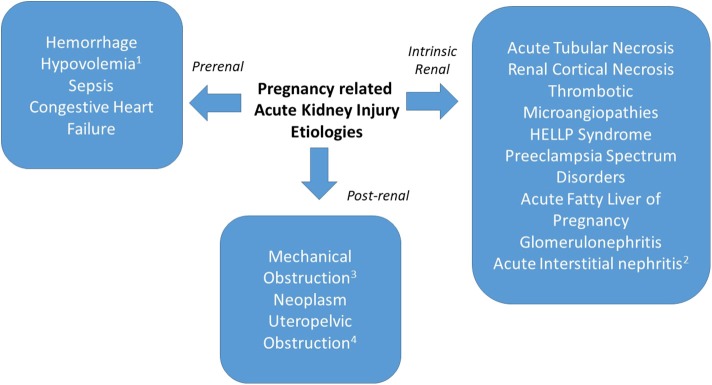
PR-AKI can have prerenal, intrinsic, and post-renal etiologies. Prerenal causes may be secondary to hemorrhage, hypovolemia (^1^from hyperemesis gravidarum), sepsis, or congestive heart failure. Intrinsic renal causes include acute tubular necrosis, renal cortical necrosis, thrombotic microangiopathy, preeclampsia spectrum disorders, acute fatty liver of pregnancy, glomerulonephritis, or acute interstitial nephritis (^2^from medication exposure). Postrenal etiologies include mechanical obstruction (^3^postsurgical), neoplasm, or uteropelvic (^4^obstruction from pregnancy).

When AKI in pregnancy occurs, one must consider both obstetric and non-obstetric etiologies. As in non-pregnant individuals, PR-AKI can be classified as having prerenal, renal and post-renal etiologies and can occur in the antepartum, intrapartum or postpartum time periods (Figure 1). The gestational age at which PR-AKI occurs can help elucidate the cause by considering the typical timing of the various obstetric complications. Additionally, a recent study by Liu et al., reported that compared to non-pregnant women, pregnant women had a 51% increased risk of developing AKI that was independent of age and clinical comorbidities, suggesting that pregnancy increases the risk of AKI (26).

**Figure 2:**
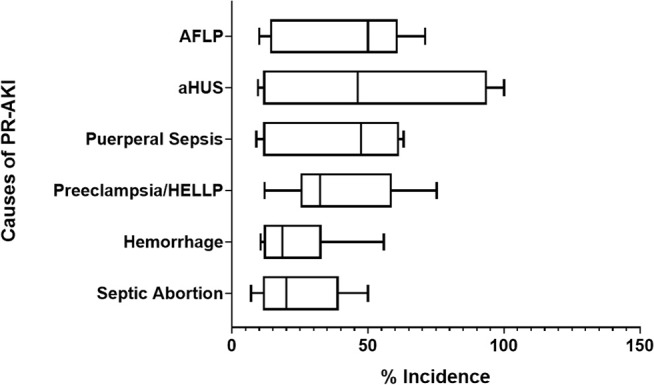
Common primary causes of PR-AKI. The most common primary causes of pregnancy related—acute kidney injury (PR-AKI) worldwide are listed along with their reported incidence.

When AKI occurs during pregnancy, it most commonly occurs in the second trimester. However, PR-AKI can occur during any trimester or in the postpartum period. Septic abortion (primarily in developing countries) can lead to AKI during the first trimester. Hypertensive disorders causing AKI can occur throughout the late second to third trimesters (27–29). As illustrated in Figure 2, the most commonly reported primary causes for PR-AKI are widespread, however these factors are often multifactorial, which can worsen the clinical scenario for the pregnant patient (27–43). PR-AKI of any etiology can be severe and in some cases can lead to ARF, or the need for renal transplantation, plasma exchange, dialysis or pharmacological treatment (3, 44). When all causes of AKI are considered, the frequency of dialysis of any duration is estimated to be 0–47% (7, 45–47) but has been reported to be as high as 97% in developing countries such as India (28). Over the past several years there has been an improvement in both maternal and fetal health outcomes due to dialysis, however more studies need to be conducted. A study conducted by Hladunewich et al., reported that after comparing 20 pregnancies from the Toronto Pregnancy and Kidney Disease Clinic and Registry with 70 pregnancies from the American Registry for Pregnancy in Dialysis Patients, chronic hemodialysis is feasible for pregnant women when managed under a strict regimen (48).

## Pregnancy Outcomes With AKI

In the United States between 1998 and 2009, 17.4% of deaths during delivery hospitalization and 31.5% of deaths among postpartum hospitalizations occurred among women with ARF of any etiology (9). Evidence suggests that hypertensive disorders of pregnancy are an important contributor to the burden of AKI in pregnancy, in particular preeclampsia and HELLP syndrome which are already associated with increased incidences of maternal and perinatal morbidity and mortality (35, 49–51). PR-AKI also has a significant impact on both maternal and fetal morbidity and mortality (26, 47). A 2017 systematic review and meta-analysis looked at maternal and fetal outcomes in cases of PR-AKI. When compared to pregnant women without AKI, those with PR-AKI had a greater likelihood of cesarean delivery, obstetrical hemorrhage, placental abruption, disseminated intravascular coagulation and an increased mortality rate (47). Women with PR-AKI also had a longer stay in the ICU, a higher incidence of stillbirth/perinatal death, lower mean gestational age at delivery (−0.70 week [95% CI −1.21 to −0.19 week]) and lower birth weight (−740 g [95% CI −1,180 to 310 g] compared to women without PR-AKI (47). Similar trends have also been reported in more recent independent studies emphasizing the impact of PR-AKI on both maternal and fetal health (26, 34).

Along with maternal complications, PR-AKI also has a significant impact on fetal morbidity and mortality (26, 47). Mortality rate has been estimated to be 23.5–38% among babies born to women with PR-AKI (45–47). There is little data regarding the long term outcome for fetuses who were exposed to maternal AKI while *in utero*. Further studies are needed to understand the long term consequences for these neonates.

While PR-AKI can be attributed to various etiologies, obstetric complications remain a significant contributor to renal injury during pregnancy. Hypertensive disorders of pregnancy, especially preeclampsia with severe features and HELLP syndrome are thought to be among the most common causes of PR-AKI. More rarely, PR-AKI is attributed to rare obstetric complications, such as atypical hemolytic uremic syndrome, thrombotic thrombocytopenic purpura and acute fatty liver of pregnancy.

## Preeclampsia

Preeclampsia is characterized by new-onset hypertension and proteinuria after 20 weeks' gestation, affects 3–5% of all pregnancies and is a major source of maternal, fetal, and neonatal morbidity and mortality worldwide (8, 20, 52). The pathogenesis of preeclampsia is believed to occur due to incomplete cytotrophoblast invasion of the uterine spiral arteries which leads to an ischemic placenta and the eventual release of inflammatory factors, immune cell activation and endothelial dysfunction (49). Long thought of primarily as a disease of hypertension, it is now well-recognized that preeclampsia is actually a multi-organ syndrome. ACOG defines preeclampsia as a systolic blood pressure of 140 mm Hg or more or diastolic blood pressure of 90 mmHg or more on two occasions at least 4 h apart after 20 weeks of gestation in a woman with a previously normal blood pressure; and proteinuria of 300 mg or more per 24 h urine collection (or this amount extrapolated from a timed collection), protein/creatinine ratio of 0.3 mg/dL or more; or a dipstick reading of 2+ (used only if other quantitative methods not available) (20). In the absence of proteinuria, preeclampsia can be diagnosed by new-onset hypertension along with the presence of a severe feature of the disease: systolic blood pressure of 160 mmHg or more, or diastolic blood pressure of 110 mmHg or more on two occasions at least 4 h apart (unless antihypertensive therapy is initiated before this time), thrombocytopenia (platelet count <100,000 × 10^9^/L), renal insufficiency (serum creatinine concentrations >1.1 mg/dL or a doubling of the serum creatinine concentration in the absence of other renal disease), impaired liver function (elevated blood concentrations of liver transaminases to twice normal concentration), pulmonary edema, new-onset headache unresponsive to medication and not accounted for by alternative diagnoses, or visual symptoms (20).

## Hellp Syndrome

HELLP syndrome is often associated with preeclampsia, as up to 20% of women with severe preeclampsia develop HELLP syndrome (45, 53). Diagnosis is typically made using the following criteria: lactate dehydrogenase elevated to 600 IU/L or more, aspartate aminotransferase and alanine aminotransferase elevated more than twice the upper limit of normal, and platelet count <100,000 × 10^9^/L (20). HELLP syndrome has a similar pathophysiology to preeclampsia as women with HELLP syndrome have abnormal placentation, immune cell activation and endothelial dysfunction (50). HELLP syndrome is also associated with increased rates of maternal morbidity and mortality (50, 54). While the mechanisms leading to preeclampsia and HELLP syndrome have not been fully elucidated, the clinical findings of edema, hypertension, proteinuria, and renal insufficiency can be explained by the changes in renal physiology that are characteristic of the disease.

## Thrombotic Microangiopathies Associated With Pregnancy

HELLP syndrome is characterized by thrombocytopenia and microangiopathic hemolytic anemia leading to end-organ injury and is considered to be the classic thrombotic microangiopathy (TMA) of pregnancy. However, there are other, less common, disorders that are also characterized by end-organ injury, thrombocytopenia and microangiopathic hemolytic anemia that defines TMA. Other TMA disorders in pregnancy include atypical hemolytic uremic syndrome (aHUS) and thrombotic thrombocytopenic purpura (TTP) (50, 55). Unlike HELLP syndrome and preeclampsia the presence of the placenta is not needed for the immediate causation of these disorders which has likely contributed to the advancement in therapies for treating aHUS and TTP. However, the difficulty in diagnosing both aHUS and TTP often leads to a delay in treatment which could have long lasting effects on both the mother and the infant [for a full review on differential diagnosis, see Gupta et al. (55)].

Dysregulation of the complement activation system is thought to be the primary cause for aHUS (56). As pregnancy challenges the immune system of the mother, the complement system is also affected which may explain why 1 in 25,000 women experience a first episode of aHUS and up to 20% of women experience an aHUS relapse during pregnancy or the post-partum period (57–59). Several risk factors for the development of aHUS associated with pregnancy, most often during the postpartum period, have been identified. There is likely an increased risk in patients who experience obstetric complications such as preeclampsia, postpartum hemorrhage, placental abruption, stillbirth or infection, nulliparity and cesarean section, or who have a personal or family history of aHUS (37, 38). However, aHUS has also been known to occur in the absence of these risk factors (36). Eculizumab, an FDA approved humanized monoclonal antibody that targets complement protein C5, has been reported to treat aHUS both in and outside of pregnancy (36, 38, 60, 61). Patients with aHUS have also benefited from plasma exchange which removes and exchanges blood plasma components from and to the circulation. Fetal outcomes tend to depend on how successful the treatment therapy was in decreasing the TMA; with women who were treated more aggressively having successful live births closer to term as opposed to women not treated or with unsuccessful therapies have a rate of stillbirth or premature infants (37).

TTP most commonly occurs due to a deficiency in ADAMTS 13 (A disintegrin and metalloproteinase with a thrombospondin type 1 motif, member 13), which is the von Willebrand factor cleaving protease (62). Unlike aHUS, pregnancy related TTP most often occurs during pregnancy as opposed to the postpartum period. It is possible for women who experienced TTP during pregnancy to have a relapse of TTP in subsequent pregnancies which has been reported to occur in up to 50% of subsequent pregnancies (63, 64). In addition, a previous pregnancy complicated with TTP increases the risk of preeclampsia in subsequent pregnancies. TTP is typically characterized by milder renal injury (serum creatinine ɤ 1.4 mg/dL) but more severe thrombocytopenia compared to aHUS (64–66). Plasma exchange is the preferred treatment method for TTP, as fresh plasma infusion will increase ADAMTS-13 levels (67). For women with a history of TTP, prophylactic aspirin and dipyridamole use has been shown to reduce the severity of relapse while also improving that pregnancy outcome (68).

## Acute Fatty Liver of Pregnancy

Acute fatty liver of pregnancy (AFLP) is a rare non-TMA obstetric emergency that if left unattended can lead to fulminant liver failure. AFLP is reported to affect between 1:7000 and 15,000 pregnant women a year and is most commonly reported to occur in the final trimester of pregnancy but can also occur during the postpartum period (69–71). A defect in the mitochondria beta-oxidation pathway has been shown to contribute to the development of AFLP in the majority of women, additional risks factors include: existing diagnosis of preeclampsia, HELLP syndrome, previous pregnancies, or currently being pregnant with a male fetus (72). Maternal and fetal morbidity can be high for patients with AFLP (69, 70), however mortality rates have significantly declined over the past few decades from 85 to 10–17% (72, 73). Diagnosing AFLP can be challenging if a patient also has preeclampsia or HELLP syndrome, but women who have at least six of the 15 Swansea criteria should be considered positive for AFLP as this criteria has been reported to have an 85% predictive value with 100% sensitivity for AFLP diagnosis (74, 75). There have been few reported cases of AFLP recurring in women with a previous pregnancy complicated by AFLP. Similar to preeclampsia and HELLP syndrome delivery is the best option to halt further organ injury, however some patients have received blood product transfusion to help improve anemia and coagulation (76–78).

## AKI in the Setting of Preeclampsia and Hellp Syndrome

Since hypertensive disorders of pregnancy are associated with an increased risk of AKI, any risk factor for hypertension can be considered as risk factors for AKI. For example, women with pre-existing renal disease are at an increased risk of preeclampsia (52), and therefore are at a higher risk of developing AKI. Preeclampsia when severe or when associated with HELLP syndrome can lead to ARF (8, 19, 20) and in the setting of HELLP syndrome alone, AKI has been reported to occur in 7–60% of patients (28, 29, 79–82). When PR-AKI occurs, it also increases the risk of other obstetric complications. AKI in the setting of HELLP syndrome and preeclampsia has been associated with placental abruption and pulmonary edema (79, 82). We have recently reported an association between PR-AKI and obstetric complications, indicating that 24% and 22% of women with PR-AKI complicated with HELLP syndrome had placental abruption and obstetric hemorrhage, respectively compared to the 13 and 11% of HELLP patients without PR-AKI (83).

Multiple studies have demonstrated that AKI is a risk factor for maternal death in patients with HELLP syndrome. In one study, AKI in the setting of HELLP syndrome carried an 11.5% maternal mortality rate (80). In several study populations, all maternal deaths in HELLP syndrome patients occurred in women with AKI, and serum creatinine levels were independent risk factors for mortality suggesting that AKI increases the already high mortality rate for women with HELLP syndrome (29, 80). Indeed this is thought to be true, as we have previously reported that in a study of women with HELLP syndrome, women with Class I HELLP (i.e., platelet levels <50,000 × 10^9^/L) had a significantly higher composite maternal mortality score (84); which upon additional analysis we found was associated with a higher risk for developing renal complications such as AKI (83). In a study that followed patients with HELLP and AKI for up to 1 year post-partum, 21.2% required dialysis (80). Some studies have reported that when compared to preeclamptic women, those with HELLP syndrome are more prone to need dialysis and remain hypertensive in the post-partum period (53, 82). The perinatal death rate associated with AKI in the setting of HELLP syndrome has been estimated to be 26–48.2% compared to the 23.5–38% seen in the absence of HELLP syndrome (46, 80, 82).

## AKI in AHUS and TTP

The incidence of AKI in pregnant women with aHUS is high which is due in part to the increased renal injury that occurs as part of the pathology of aHUS, independent of pregnancy status or gender (85). AKI is often severe enough to require immediate dialysis and/or plasma exchange. A study by Bruel et al. found no improvement in renal injury after plasma exchange among women with pregnancy associated aHUS (36). However, several studies have reported improvements in renal outcome and reduced the need for dialysis when eculizumab is added to the treatment plan (37, 38).

For TTP patients that do develop AKI, it is not as severe as seen in other TMA disorders and the reported incidences are not as high (67, 86, 87). While it has been reported that TTP is a risk factor for the development of CKD, this risk is independent of AKI (67). It appears that one of the greatest risk factors for postpartum renal injury is the misdiagnosis among TMA disorders. A recent retrospective study by Meibody et al., reported that among nine French clinical centers, of the 105 postpartum AKI women admitted over a 5-year period of time, as none of the patients were initially diagnosed aHUS (*n* = 10) or TTP (*n* = 4) (87).

## AKI and AFLP

Several studies have reported AKI or renal insufficiency in >50% of patients with AFLP (73, 76, 88, 89). One study from the Netherlands reports that of six women with AFLP who died, 67% (*n* = 4) of the patients had renal failure as a maternal complication and 50% of the 12 surviving AFLP patients had renal injury (71). The renal injury in AFLP has been proposed to possibly occur due to microvesicular fat in the kidney as a result of inhibition of beta-oxidation of renal fat (90, 91). However, studies have reported that within 6 months of delivery renal injury, even for patients with ARF, has been reversed (88, 92, 93).

## Long Term Consequences of PR-AKI

It was initially thought that the organ dysfunction that occurs with preeclampsia and HELLP syndrome reversed after delivery. There is now increasing evidence that a pregnancy complicated with preeclampsia and/or HELLP syndrome is a risk factor for chronic or future renal disease in addition to cardiovascular disease. Recent studies report that preeclampsia is associated with an increased risk of remote cardiovascular disease and ESRD (94). In the past, AKI was also considered to be a completely reversible syndrome, however, in recent years, several studies have indicated that AKI may increase the risk of developing CKD, incurring continued kidney damage, or requiring dialysis even after delivery (28, 47, 95). Estimates of long-term prognosis after AKI in pregnancy are limited by inconsistent definitions and follow-up duration.

A meta-analysis on PR-AKI reported that 2.4% (95 CI 1.3% to 4.2%) of women with AKI during pregnancy progressed to ESRD and needed long-term dialysis (47). In a study that followed patients with HELLP and AKI for up to 1 year post-partum, 21.2% required dialysis (80). Some studies have reported that when compared to preeclamptic women, those with HELLP syndrome are more prone to need dialysis and remain hypertensive in the post-partum period (53, 82). Complete recovery of renal function has been estimated to occur in 82.7–89.4% of patients, however long-term data regarding dialysis in women with a history of AKI in pregnancy is lacking (46, 80). Although the absolute risk of ESRD after preeclampsia is low, preeclampsia in one or more pregnancies is a risk factor for development of long term renal dysfunction (94). There are few long-term outcome studies reporting on the incidences of CKD or renal function in women with PR-AKI in the setting of preeclampsia or HELLP syndrome. Some studies report that renal recovery is estimated to occur in up to 74.4% of affected women (80), while others report that HELLP does not worsen the long-term renal prognosis relative to women with PR-AKI for other reasons (81). Even when resolution of kidney injury does occur, normal renal function is not always restored in the post-partum period (96). Women with a history of HELLP syndrome also have increased incidences of persistent renal pathology in the post-partum period. A recent study by Ye et al. reported that among six HELLP patients who had a renal biopsy 2–6 weeks postpartum due to persistent AKI symptoms the most common lesion was acute tubular necrosis (ATN) (97). While these women did have a complete renal recovery it was noted that HELLP patients with an additional TMA diagnosis in addition to the ATN developed chronic renal dysfunction, suggesting that TMA when coexisting with ATN may potentiate chronic kidney disease.

For patients with a history of aHUS and AKI, one study reported that several years (7.2 ± 5.2 year) following a pregnancy complicated with aHUS 53% of patients had progressed to ESRD (36). Poor renal outcome in patients with pregnancy associated aHUS were reported in other studies where 21–36% of patients either developed CKD, received a renal allograft, were on dialysis or developed ESRD (37, 57). For women treated with eculizumab during pregnancy associated aHUS there have been no reports of any of the 17 women progressing to ESRD or needing dialysis after pregnancy (38).

## Translational of PR-AKI With Experimental Animal Models

The mechanisms by which AKI can lead to CKD have not been fully elucidated and the relationship between renal disease during pregnancy and hypertensive pregnancies is not well-defined. Therefore, it is important to utilize experimental models to study possible mechanisms and physiological pathways to help us better understand the relationship between hypertension during pregnancy, renal injury and the progression to chronic renal disease. In clinical settings, AKI during pregnancy is often due to preeclampsia, so it stands to reason that most experimental animal models of renal injury during pregnancy are preeclampsia (PR) or pregnancy-induced hypertension (PIH) models (Table 2). While these current models have been used to study PR-AKI, a true animal model for the disorder does not exist. Studies in our lab have developed an animal model of PR-AKI and have found that bilateral renal ischemia on gestational day 18 in the pregnant rat leads to reduced GFR, significant renal fibrosis and hypertension. Importantly, our preliminary data does indicate that this single episode of AKI does lead to the development of CKD by 3 months postpartum.

**Table 2:** Animal models with hypertensive pregnancies and commonly reported renal injuries.

**Model name**	**Host**	**Injury type**	**Renal injury characteristics**	**References**
Dahl salt sensitive rat (Dahl-S)	Rat	Spontaneous and genetic	Antepartum—proteinuria, kidney injury via pathological scores, increases in glomerular area and diameter Postpartum—Proteinuria, increased neprhin excretion, kidney injury via pathological score, increased infiltration of renal T cells	(98, 99)
Renin-angiotensin system (RAS)	Mouse	Transgenic	Antepartum—increased albuminuria, kidney injury via pathological scores, decreased perfusion of glomeruli, glomerular endotheliosis, decreased number of erythrocytes in the capillary lumen of glomeruli	(100)
NG-nitro- L-arginine methyl ester (L-NAME)	Rat/mouse	Chemical	Antepartum—Proteinuria, increased blood urea nitrogen, increased serum creatinine, kidney injury via pathological scores	(101)
Reduced uterine perfusion pressure (RUPP)	Rat	Mechanical obstruction of ovarian and uterine arteries	Antepartum—decreased GFR, increased renal sympathetic nerve activity, decreased superoxide dismutase, proteinuria, reduced renal plasma flow Postpartum—decreased GFR	(102–105)

## The Link Between AKI and Neurocognition

**Figure 3:**
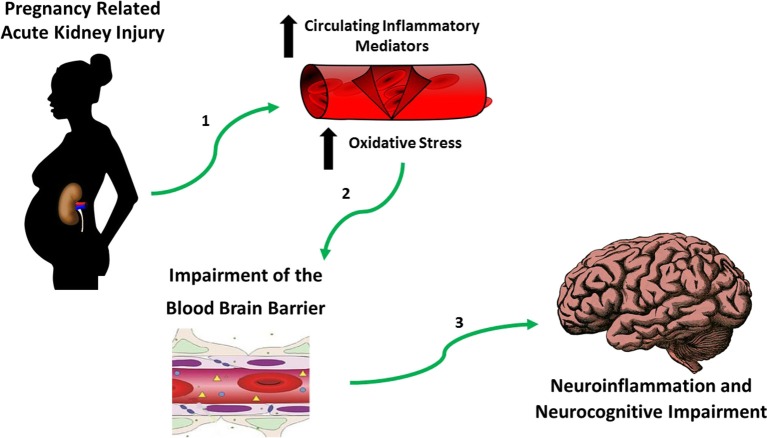
Hypothesis establishing the link between PR-AKI and neurocognitive impairment. Based on published studies and our own preliminary work we believe that in the setting of pregnancy related-acute kidney injury (PR-AKI), (1) there's an increase in circulating inflammatory mediators and oxidative stress which (2) damages the blood brain barrier^a^ which allows for (3) neuroinflammation to take place eventually contributing to neurocognitive impairment. ^a^Review Varatharaj and Galea (111).

It is important to recognize that neurocognitive impairment is a key feature associated with both AKI and CKD (106). Both hypertensive pregnancies and CKD are independently related to cognitive dysfunction (107–110), it is important not to overlook the possible impact that they may have on the patient with a history of PR-AKI. The available data has led us to propose the following theory (Figure 3) suggesting that the increase in circulating inflammatory mediators (112) and oxidative stress (113) due to PR-AKI leads to impairment of the blood brain barrier during pregnancy which in turn contributes to neuroinflammation and neurocognitive impairment in the postpartum period and later in life; a situation that is only worsened in the presence of a hypertensive pregnancy.

In summary, PR-AKI is a severe obstetric complication that can have devastating maternal, fetal and neonatal effects. The rising incidence of PR-AKI and its association with the hypertensive and TMA disorders of pregnancy have been established. However, questions remain regarding the precise pathophysiologic mechanisms leading to renal injury and subsequent sequelae. Development of a uniform diagnostic criteria and refining of animal models will help advance our knowledge and understanding of the clinical implications of acute renal injury during pregnancy. Additionally, as work continues to identify the best nutritional diets, health plans and safe therapeutics to prophylactically administer during pregnancy to women with hypertensive and TMA disorders (114–116) we can also hope to see a decrease in fetal and maternal mortality and morbidity.

## Author Contributions

JS and AG contributed to the writing and editing of this manuscript. SN contributed to the concept and editing of this manuscript. KW contributed to the concept, writing, and editing of this manuscript.

### Conflict of Interest

The authors declare that the research was conducted in the absence of any commercial or financial relationships that could be construed as a potential conflict of interest.
